# Towards improved screening of toxins for Parkinson’s risk

**DOI:** 10.1038/s41531-023-00615-9

**Published:** 2023-12-19

**Authors:** Ling Shan, Harm J. Heusinkveld, Kimberly C. Paul, Samantha Hughes, Sirwan K. L. Darweesh, Bastiaan R. Bloem, Judith R. Homberg

**Affiliations:** 1 Department Neuropsychiatric Disorders, Netherlands Institute for Neuroscience, an Institute of the Royal Netherlands Academy of Arts and Sciences, Amsterdam, the Netherlands; 2 Centre for Health Protection, National Institute for Public Health and Environment (RIVM), Bilthoven, The Netherlands; 3 Department of Neurology, David Geffen School of Medicine, University of California, Los Angeles, Los Angeles, CA USA; 4 A-LIFE Amsterdam Institute for Life and Environment, Section Environmental Health and Toxicology, Vrije Universiteit Amsterdam, De Boelelaan 1085, 1081 HV Amsterdam, The Netherlands; 5 Department of Neurology, Donders Institute for Brain, Cognition and Behaviour, Radboud University Nijmegen Medical Centre, Nijmegen, The Netherlands; 6 Department of Cognitive Neuroscience, Donders Institute for Brain, Cognition and Behaviour, Radboud University Nijmegen Medical Centre, Nijmegen, The Netherlands

**Keywords:** Parkinson's disease, Research data, Risk factors

## Abstract

Parkinson’s disease (PD) is a chronic, progressive and disabling neurodegenerative disorder. The prevalence of PD has risen considerably over the past decades. A growing body of evidence suggest that exposure to environmental toxins, including pesticides, solvents and heavy metals (collectively called toxins), is at least in part responsible for this rapid growth. It is worrying that the current screening procedures being applied internationally to test for possible neurotoxicity of specific compounds offer inadequate insights into the risk of developing PD in humans. Improved screening procedures are therefore urgently needed. Our review first substantiates current evidence on the relation between exposure to environmental toxins and the risk of developing PD. We subsequently propose to replace the current standard toxin screening by a well-controlled multi-tier toxin screening involving the following steps: in silico studies (tier 1) followed by in vitro tests (tier 2), aiming to prioritize agents with human relevant routes of exposure. More in depth studies can be undertaken in tier 3, with whole-organism (in)vertebrate models. Tier 4 has a dedicated focus on cell loss in the substantia nigra and on the presumed mechanisms of neurotoxicity in rodent models, which are required to confirm or refute the possible neurotoxicity of any individual compound. This improved screening procedure should not only evaluate new pesticides that seek access to the market, but also critically assess all pesticides that are being used today, acknowledging that none of these has ever been proven to be safe from a perspective of PD. Importantly, the improved screening procedures should not just assess the neurotoxic risk of isolated compounds, but should also specifically look at the cumulative risk conveyed by exposure to commonly used combinations of pesticides (cocktails). The worldwide implementation of such an improved screening procedure, would be an essential step for policy makers and governments to recognize PD-related environmental risk factors.

## Introduction

Parkinson’s disease (PD) is one of the most prevalent neurodegenerative diseases in the world. It is characterized in part by motor disturbances, including tremor rigidity and bradykinesia, which result from loss of dopaminergic neurons in the substantia nigra^1,2^. PD is a complex multi-system disorder with a prodromal phase that includes gradually emerging non-motor symptoms, rapid eye movement sleep behaviour disorder symptoms, gastrointestinal and autonomic dysfunctions, which can precede the clinically based diagnosis by many years, perhaps even decades^2–4^. Since the first description of the disease by Dr. James Parkinson in 1817^5,6^, the disease has become much more prevalent. Between 1990 and 2016, the total PD population increased from 2.5 to 6.1 million individuals worldwide^7^. There are several potential factors contributing to the increase in the burden of PD, including the growing size of the aging population, a longer survival with PD, lifestyle choices like physical inactivity and certain dietary habits, and exposure to toxins from industrialization. Among the modifiable risk factors, individuals have least control over exposure to industrial (by) products^8,9^. A growing body of evidence suggests that exposure to these products, including pesticides, solvents heavy metals, micro plastics and air pollution (collectively called toxins), is at least in part responsible for this rapid growth.

A particularly worrying insight is that the current screening procedures to test for a possible (neuro)toxicity of specific compounds applied internationally under various regulations, are inadequate to assess specific PD-risks^10–14^. In order to decrease the possible risk of PD following exposure to these toxins, an improved and systematic screening and regulation process is essential. Here, we propose that well-controlled and multi-tiered toxin screening studies are an essential step to provide actionable data for policy makers and governments to recognize PD-related environmental risk factors. To substantiate this proposition, we review epidemiological and neuropathological/toxicological data, acknowledging that such studies may lack sufficient detail with regards to the types of toxins (epidemiological data), dose, duration of exposure, and representative genetic background of the tested populations. In this review, we tried to focus on pesticides, which show the strongest correlation to PD risks, and which have also been well studied in vitro and in vivo.

We further provide a critical summary of the current knowledge of the most widely studied PD-related pesticides, including rotenone and paraquat, on PD-like features in experimental animal studies. Based on this information, we identify important knowledge gaps that will help to design an improved screening procedure. We will outline that such an improved strategy could test the possibility that some currently used toxins, despite having passed current screening procedures, might yet reveal a heightened risk of causing PD. We conclude by making recommendations as to how improved experimental models offer a better insight into the risk of neurotoxicity of both single chemicals and mixtures of chemicals. Use of such improved experimental models can offer a better insight into the risk of neurotoxicity to the substantia nigra and consequently the risk of PD in humans.

## Human studies

### Epidemiologic evidence: increased risk of PD in individuals exposed to pesticides

Epidemiologic studies support the association between pesticides and PD risk^15–17^. A meta-analysis of 46 studies from around the world reported a summary risk ratio of 1.6 [95% confidence interval (CI) 1.4-1.9] pesticide exposure (ever versus never)^18^. The risk ratio is consistent with a family based study reporting that individuals exposed to pesticides were significantly more likely to develop PD than their unaffected relatives (odds ratio 1.61, CI = 1.13–2.29)^19^. The French AGRICAN cohort study revealed that, after adjusting for sex, age, educational backgrounds and many other factors, there is higher PD risk with ever use of specific pesticides such as rotenone (risk ratio of 1.57, CI = 1.08-2.29) and paraquat (risk ratio of 1.43, CI = 1.17–1.75) on various crops^20^. It should be noted that a recent study also showed that exposure to acute and accidental exposure to high doses of pesticides is associated with Dream-Enacting Behaviours, which is a relatively specific prodromal symptom of PD^21^. These associations may reflect causal effects of pesticides on the risk of PD, although other explanations (e.g., residual confounding) cannot be ruled out, in particular because most studies were based on ecological pesticide exposure measurements. Furthermore, there are variations in the findings of these studies. For instance, rotenone is a pesticide and a piscicide (toxic to fish), whose mode of action is as an inhibitor of mitochondrial complex I that freely crosses the blood brain barrier. A case-control study, based on professional pesticide applicators who are able to reliably report pesticide usage, demonstrated a significant 2.5-fold increase risk of PD in those who had been exposed to mixed and applied rotenone (CI = 1.3, 4.7)^22^. Within the same study, paraquat, a blood brain barrier permeable herbicide that is commercially available since 1961 and is one of the most widely used pesticides in the world^23^, was associated with a 2.5-fold increase risk of PD (CI = 1.4, 4.7)^22,24,25^. A small early study based on 102 PD patients and 84 controls and self-reports on pesticide exposure demonstrated an odds ratio for rotenone of 10.0 (CI = 2.9–34.3)^26^. An increased all-cause mortality risk in PD patients with occupational exposure to pesticides was reported in a prospective cohort with 150 idiopathic PD patients enroled from 2008 to 2013^27^. Another longitudinally followed two PD patients cohorts on an average of 5 years and 2.7 years and showed that specific pesticide exposure is not only be relevant for PD onset symptoms but also PD progression phenotypes including faster PD motor and non-motor decline^28^. Other studies, however, found no association between paraquat and PD^29,30^. These inconsistent findings may be due to multiple factors, for instance the limited sample size or inconsistencies in exposure classification/exposure misclassification or, perhaps most importantly, individual differences in genetic background. Indeed, many established PD genes are known to interact with pesticides and other environmental toxins^31^. If a genetic factor modifies the influence of exposure, then exposed populations with more variant carriers would be better suited to detect associations. However, not accounting for such factors can also result in varying consistency of the marginal associations^32^. For instance, a study reported that individuals who lack an active copy of the gene encoding for the xenobiotic metabolic enzyme glutathione-S-transferase T1 (*GSTT1*) have a higher risk of PD following paraquat exposure^33^. Two studies have also suggested that dopamine transporter gene variants modify paraquat exposure risk^34,35^.

### Neuropathological evidence: organochlorine pesticides higher in PD brains

Neuropathological studies provide further corroboration that pesticide exposure is associated with a higher PD risk^22,36^. Dieldrin, an organochlorine pesticide, was detected significantly more often and at higher levels in (6 out of 20) PD brains than in age and sex matched (1 out of 7) Alzheimer’s brains and in (0 out of 14) control brains^37^. Furthermore, the levels of the gamma isomer of hexachlorocyclohexane were significantly higher in PD brain tissue compared to AD and controls^38^. Organochlorine pesticides, especially heptachlor epoxide, have been heavily used in pineapple fields in Hawaii after World War II and were banned in 1988. The contamination was traced in the groundwater^39^ as well as milk supply^40,41^. The prevalence of Lewy pathology was significantly increased in the presence of organochlorines including heptachlor epoxide, hexachlorobenzene and α-chlordane in 705 brains from the Honolulu-Asia aging study^42^. It should be noted that most of the organochlorine compounds are now banned due to their high persistence, bioaccumulation and carcinogenesis^43^. In addition, the detection of organochlorine provides an interesting piece of evidence but it is rather circumstantial, because there are many more chemical substances involved in PD that cannot be detected in human brain samples related to kinetics.

### Open questions from epidemiological and neuropathological studies

In summary, the above reviewed epidemiological and neuropathological studies demonstrate that pesticides and heavy metals have been linked to the development of PD. However, these studies have some limitations. Firstly, high-quality epidemiological studies with data that are sufficiently detailed to identify essential aspects of exposure (such as type of toxin, dose, duration of exposure and genetic background of the tested populations) are lacking. Furthermore, and importantly, the epidemiological and neuropathological studies are mostly retrospective, which precludes inference of causality of the observed associations. This makes it challenging to integrate such data into risk assessments. It will take many years and even decades to have evidence-based studies in place.

### Improved neurotoxin screening is urgently needed

Rapid growth in the chemical industry over the past 50 years and integration of manufactured chemicals into nearly every aspect of industry means that tens of thousands of chemicals are commercially available and widely used worldwide^44^. For instance, 453 different pesticide active ingredients are registered for use in the European Union and over 1000 in the United States^45^, however, for the majority of exposures, adequate information pertaining to most health risks, including PD, is unavailable. This is despite decades of findings from epidemiologic studies that have linked general pesticide use, as well as rural living and occupational farming, to an increased risk of PD, the vast majority of specific pesticides widely used in agriculture have not been assessed for off-target neurotoxicity and their possible contribution to PD. Most epidemiologic studies assess exposure using a candidate approach, selecting specific targeted agents or use a general, non-specific pesticide summary measure. For instance, the epidemiologic finding that PD is associated with paraquat^46^ triggered decades of experimental and mechanistic studies on the topic^47^. It is increasingly clear that a much wider screen of commercially applied pesticides in relation to PD is needed. High-throughput screening combined with record-based untargeted exposure approaches and exposomic human studies provide a means to more comprehensively investigate the vast number of pesticides and chemicals introduced by industry and to help to prioritize agents for more in depth targeted epidemiologic and animal studies.

### The way forward

**Fig. 1:**
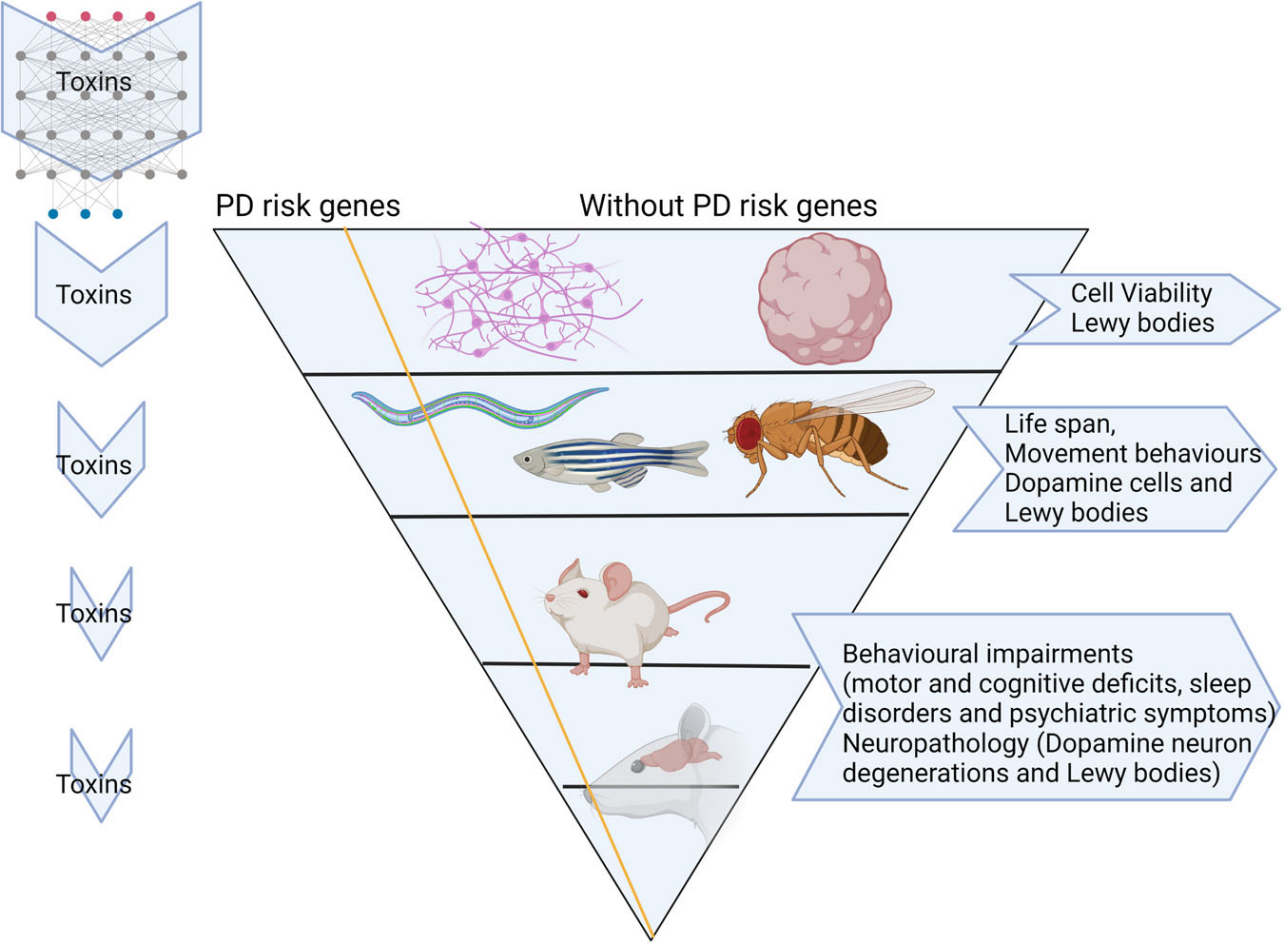
There are four tiers of screening including in silico, in vitro, in vivo (*Caenorhabditis elegans*, *Drosophila melanogaster* and Zebrafish) and more complex animal models for in vivo screening (rodents: mice and rats). The shape of the inverted triangle refers to the likelihood of a specific tier being used – greatest for Tier 1, lowest for Tier 4. Tier 1: In silico techniques: To help the prioritization of agents for more in depth research, machine learning and artificial intelligence, combined with techniques such as QSAR and molecular docking, can be used to screen adverse outcome pathways covering the environmental toxins (single or mixture) that link the molecular targets or pathways^145,146^. Those pathways are not limited to mitochondrial complex dysfunction, impaired proteostasis, neuroinflammation and degeneration of dopaminergic neurons of the substantia nigra. Tier 2: In vitro techniques: Cell-based assays, such as the dopaminergic cell lines (e.g., SH-SY5Y cell line or Lund human mesencephalic (*LUHMES*) neuronal cells), or more complex human stem cell models, or human-induced pluripotent stem cell (hiPSC)-derived neuronal cultures and brain organoid, are employed to further test toxin effects. Tier 3: Simple organism models: *C. elegans*, *D. melanogaster, and Danio rerio are used to screen toxin effects*. These models provide excellent whole-organism high-throughput screening models for both single chemicals and mixtures. Previous work has proven their value in the assessment of pesticide-induced (developmental) neurotoxicity. Tier 4: In vivo Rodent test: Taking the advantage of mice and rats used in the regulatory required toxicity studies, we can generate high quality, neuropathological and behaviour data for toxins, specifically addressing PD. (Created with BioRender.com).

Generally, toxins display their PD risk after a relatively long period of exposure. In addition, because toxins are contained in nature for a long period of time, it may take years before existing legislative actions regulating, or banning, these highly pervasive neurotoxic substances. Importantly, current neurotoxicity screens are inadequate to examine PD risks^10^. The occurrence of acute behavioural changes in animal models will not necessarily predict a long-term effect on the risk of PD, a disease that typically becomes manifest clinically only after a long period of time (and presumably after a long time of exposure to environmental toxins). A conceivable explanation is that the process to deplete 60–80% of dopaminergic neurons in the nigrostriatal pathway of PD patients—which is the threshold beyond which clinically discernible symptoms start to emerge—likely takes decades^1^ together with aging. To reduce the frequency and burden of PD, a structuralized screening of potentially neurotoxic compounds is urgently needed. As we mentioned previously, a multi-tier approach including In silico, In vitro, single organism models and in vivo rodent experiments is required. Since most well-controlled epidemiological and neuropathology studies are predominantly retrospective, a combination of PD risk gene factors and variable pesticides should be tested thoroughly in the laboratory in both in vitro and in vivo systems prospectively. We therefore propose a multi-tier toxin screening for both familial and sporadic PD (Fig. 1) that covers scales of biological organisation.

### The next generation of neurotoxin screening in the animal tests

Neurotoxicity testing for regulatory purposes is partly based on in vivo animal studies, which do not include PD-specific behaviour tests^48,49^, nor PD-related neuropathology studies such as midbrain dopaminergic cell counts or α-synuclein histology^10^. Well-controlled multi-tier toxin screening studies including in silico, in vitro and in vivo models for PD in the laboratory will therefore allow us to examine PD risks more specifically (Fig. 1). These tiers of studies are also more feasible and less expensive than the aforementioned PD epidemiological and neuropathological studies. Furthermore, they allow for investigation of causal relationships between toxin exposure, PD neuropathology and behavioural outcomes, and can provide clues for experimental mechanistic studies that may suggest therapeutic strategies. The inclusion of in silico and in vitro models and lower (in) vertebrate organisms such as the fruit fly *Drosophila melanogaster* and the nematode worm *Caenorhabditis elegans* have many advantages over mammalian animal testing, including short generation times, being cheap to cultivate and favour high throughput screening applications without ethical constraints. On the other hand, rodent animal models are indispensable due to longer term physiological effects as well as behavioural readouts^50^. Together, the different scales of biological organisation will prove valuable to unravel the toxicity pathways that drive PD progression.

## Animal studies

### The Rotenone PD model

- Neuropathological: mainly selective nigrostriatal degeneration, increased number of Lewy body-like inclusions, increased number of microglia, induction of microglial activation.
- Neurochemical: lower level of dopamine in the striatum, increase in neuroinflammation markers such as tumour necrosis factor-α (TNFα) and interleukin 1 beta (IL-1β).
- Behavioural symptoms: motor symptoms, including ipsilateral rotation behaviour, postural instability, rigidity and bradykinesia; non-motor symptoms, hyposmia, gastrointestinal dysfunction, sleep disturbances, circadian dysfunction.

Compelling in vivo animal studies identified rotenone as a risk factor for PD. Furthermore, similar to the neuropathological presentation of PD patients, noradrenergic neurons are lost in the locus coeruleus while in the ventral tegmental area dopaminergic neurons are relatively spared in rotenone exposed rats^51^. In addition to these neuropathological findings, non-motor symptoms including hyposmia, gastrointestinal dysfunction, sleep disturbances and circadian dysfunction were found in rotenone rodent models (reviewed in^52^).

Rotenone also has been found to induce microglial activation and increase the number of microglia in rodent models^53–57^, with pro-inflammatory factors tumour necrosis factor-α (TNFα) and interleukin 1 beta (IL-1β) upregulated in the rotenone lesioned rodent model^56^.

Furthermore, rotenone as an environmental risk factor appears to enhance genetic susceptibility to PD symptoms. For instance, 6-month old E46-mutated α-synuclein transgenic rats display a significantly greater reduction of striatal dopamine terminal density and more pronounced motor deficits compared to a similar rotenone challenge in wild type rats^58^. Increased levels of α-synuclein and phosphorylated α-synuclein as well as mild motor deficits have been observed in rotenone challenged α-synuclein mutation (A53T) over-expression mice, whereas the transgenic mice administered vehicle had no changes^59^.

### The paraquat-induced PD model

- Neuropathological: degeneration of nigral dopaminergic neurons, increase in number of activated microglia.
- Neurochemical: increased reactive oxygen species production.
- Behavioural symptoms: impaired locomotor activity, non-motor symptoms including shorten life span.

Paraquat, also induced selective degeneration of nigral dopaminergic neurons and an increase in the number of activated microglia in animal models^60–62^. The dopaminergic neuronal loss requires repeated chronic exposure to a low dose of paraquat to increase the reactive oxygen species production in the brain as well as to activate microglia^60,61,63–65^. Exposure to paraquat has been found to promote tyrosine phosphorylation of parkin and to silence Wnt/Wingless signalling in the dopaminergic SH-SY5Y cell line^66,67^. In addition, paraquat induces histone H3 acetylation and apoptosis in an in vitro model of PD^68^. Together, these findings are consistent with those demonstrating that paraquat induces the production of reactive oxygen species, which serve as a significant regulator of N6-methyladenosine methylation of circular RNAs^69^. Toxins triggering reactive oxygen species production in the brain are therefore included in the sections of future investigations as an important readout of toxins exposure (the way forward (Fig. 1)).

Paraquat exposure does not only occur in isolation, but also in combination with the fungicide maneb (manganese ethylene-bis-dithiocarbamate). In animal models, the combined chronic administration of paraquat and maneb, more so than administration of only one of the chemicals, was found to cause nigro-striatal degeneration, microglial activation, lipid peroxidation and motor deficits that are manifested as hunched posture and a decline in locomotor activity^70–72^,which could be alleviated by co-administration of L-DOPA^73^ This points towards an additive or synergistic effect. Gastric co-administration of subthreshold dose of lectins and paraquat can induce a range of PD-like neurodegenerative processes and pathological changes, including misfolded α-synuclein in the dorsal motor nucleus and substantia nigra, a loss of dopaminergic neurons in the substantia nigra, and Parkinson-related behavioural deficits that are responsive to L-DOPA therapy^74^. We therefore also suggest in the way forward (Fig. 1) to study not only a single toxin from the environment, but also a mixture of toxins in relation to the PD risk.

### Alternative models

Non-mammalian models are becoming more frequently used in PD studies. The genes that result in monogenic forms of PD have been identified in the nematode *C. elegans*^75^*, fruit fly Drosophila* and the zebrafish *Danio rerio*^76^. and all species have orthologs for parkin^77–80^, DJ-1^77,81–83^, and LRRK2^84–90^, and it was collectively found that these genes enhance mitochondria toxicity and increase the vulnerability of dopaminergic neurons to rotenone. This has resulted in the use of these organisms to further understand the cellular and molecular mechanisms of disease progression, as well as the evaluation of toxins on neurodegenerative disease. Building on these observations, many compounds are currently tested in in vitro or in vivo models to identify therapeutic agents for PD treatment^72,91,92^. To this end, we have included in the section of future investigations that toxins (the way forward (Fig. 1)) and their possible interactions with genetic PD risk factors (from familial PD and sporadic PD genome-wide association studies (summarized in Table 1)) need to be investigated.

**Table 1:** Targets of genetic factors associated with Parkinson’s disease genetic risk factors interacted with environmental toxicant in Parkinson’s disease^175^.

Gene	Locus	Nomenclature	Targets	Toxicant	Exposure
SNCA	4q21–22	PARK1	Mitochondrial dysfunction	Rotenone	Household
Parkin	6q25–27	PARK2			
PINK1	1p25–36	PARK6		Organochlorines	Agriculture and mosquito control (banned in the USA)
LRRK2	12p11.2q13.1	PARK8			
ATP13A2	1p36	PARK9		Pyrethroids	Household and agricultural
HTRA2	2p12	PARK13		Manganese	Occupational, ground water, dietary
PLA2G6	22q13.1	PARK14		Iron	Occupational, ground water, dietary
CHCHD2	7p11.2	PARK22			
VPS13C	15q22.2	PARK23			
SNCA	4q21–22	PARK1	Oxidative stress	Paraquat	Agriculture
DJ1	1p36	PARK7			
LRRK2	12p11.2q13.1	PARK8		Organochlorines	Agriculture and mosquito control (banned in the USA)
ATP13A2	1p36	PARK9			
				Organophosphates	Agriculture

#### Caenorhabditis elegans

The nematode worm *C. elegans* phenocopies hallmarks of PD^93–97^. While sequencing of *C. elegans* genome did not find orthologues for α-synuclein, it is possible to study the effects of different α-synuclein variants by overexpressing these in the nematodes without having to consider the effects of background α-synuclein expression^98^. Expression of the human PD related protein in nematodes shows age-dependent aggregation, and when expressed in dopaminergic neurones results in neurodegeneration^99,100^. It is important to note that those *C. elegans* expressing α-synuclein have a normal lifespan^101,102^but are more sensitive to toxin exposure and dopamine neurone degeneration^102^. The transparency of the nematode enables easy visualization of fluorescent reporters, such that tagging the α-synuclein to GFP or YFP has shown that the resulting aggregates resemble the Lewy body-like inclusions in human PD and lead to increasing toxicity with age^94,101^, but has also enabled the identification of neuroprotective genes^94,103^ and compounds that affect α-synuclein aggregation^101,104^. Strikingly, as dopaminergic neurones are not essential for life in *C. elegans*^105^, it is possible to dissect the link between onset of PD and lifespan as determined by aging and to this end, work has shown that disease onset can be delayed by interventions that increase lifespan^102,106^.

The interaction between the established PD genes and environmental toxins has been explored using *C. elegans*, usually as single mixtures^77,84,107–109^. In nematodes, exposure to rotenone causes a reduction in dopaminergic neurones, altered mitochondrial biogenesis and an increase in α-synuclein aggregations^107,110,111^. *C. elegans* over-expressing α-synuclein mutation A53T showed increased sensitivity to rotenone with reduced viability, lower oxygen consumption and visible α-synuclein aggregations compared with non-transgenic controls^77^. Paraquat exposure also has a detrimental effect on nematodes, with a significant change to mitochondrial bioenergetics and DA neurodegeneration^111,112^. In addition, changes in neuronal morphology can be quantified and then linked to changes in behaviour, providing an indicator of neuronal damage^113–117^.

#### Drosophila melanogaster

Despite being a simple model, *D. melanogaster* shows complex behaviours such as walking, climbing and flying^118,119^. The fruit fly serves as models of human disease, notably neurodegenerative diseases^120,121^ and, to identify novel drug entities^122^. Importantly, the presence of a series of cells that act as a blood-brain barrier, subperineurial glia which functions in a similar fashion to that in mammals^121,123^, provides many opportunities to study how pesticides disrupt the formation of the blood-brain barrier and impact neurodevelopment and neurodegeneration.

While in most cases no *D. melanogaster* orthologs of a specific PD-linked gene exist, the flies can be readily genetically modified to mis-express the human gene either in its full length or mutant form^118^, with mutated α-synuclein linked to familial PD, A30P and A53T being expressed in the flies to enable modelling of PD^119^. Flies exposed to sub-lethal yet chronic doses of rotenone display locomotor defects^124^ as well as, mitochondrial and dopaminergic neurone dysfunction^125^. Similarly, chronic low doses of paraquat shorten the life span and impair locomotor activity of fruit flies^91,126–128^. Interestingly, recent data has suggested that young flies are more tolerant to paraquat compared to older flies^129^, suggesting that more exploration is required to test the impact of pesticides at different life stages in different genetic background.

#### Danio rerio

Another relevant model is the zebrafish, *Danio rerio*. Zebrafish embryos (non-animal) develop major tissues, including the brain and central nervous system, by five days post fertilization, after which they are considered animals^130,131^. The evolutionary conservation of processes involved in the development of the brain, the presence of most human-relevant neurotransmitter pathways and structures, as well as the development of a blood–brain barrier^132^, together supports the translational relevance of this model species and the potential to predict effects in mouse and humans^133,134^. The pathological analysis of PD using zebrafish as a model has been reviewed in several reviews^76,135,136^, with. transgenic expression of PD risk genes with certain toxin exposure triggering key morphological, physiological, and biochemical defects.

The readouts of pesticide exposure in zebrafish involve behavioural deficits (i.e. reduced spontaneous swimming and velocity), a shorter lifespan, degeneration of dopamine cells and the formation of α-synuclein accumulations like-structures in the brains of these animal models^137,138^. Similar to other model species, zebrafish exposed to rotenone results in locomotor defects^139^ and a reduction in dopamine^140^. Zebrafish exposed to paraquat display an increase in oxidative stress^141^, mitochondrial dysfunction^142^ and a reduction in dopamine neurons^139^. In adult zebrafish, cognitive defects were apparent^143,144^ (Box 1).

Box 1 A multi-tiered screening system for neurotoxicity As illustrated in Fig. 1, we propose a screening approach that successively integrates model applications with increasing complexity. These models allow for the assessment of environmental factors and their possible interactions between genetic PD risk factors or sporadic (unknown genetic cause) PD. We propose the following approach:

### Tier 1: in silico research

Firstly, we must initially consider exposure to a wide range of chemicals. This includes starting with product registration lists (e.g., all registered pesticide active ingredients and products), public databases of commercial use when available to assess prevalence of potential exposure (e.g., California’s Pesticide Use Report database to determine how widespread pesticide use is), and toxicogenomics databases (e.g., Comparative Toxicogenomics Database). Machine learning and artificial intelligence can be used to screen adverse outcome pathways covering the environmental toxins (single or mixture) that link the molecular targets or pathways^145,146^ such as mitochondrial complex dysfunction, impaired proteostasis, neuroinflammation and degeneration of dopaminergic neurons of the substantia nigra to parkinsonian motor deficits. There are several available testing strategies including Quantitative Structure-Activity Relationship (QSAR)^147,148^, molecular docking^149^ and adverse outcome pathway analysis^150^. The goal is to start with a wide, comprehensive list of chemicals with routes of exposure to humans, to follow with in vitro high-throughput screening to help prioritize agents for more in depth research^151^.

### Tier 2: in vitro research

Conduct experiments with cell-based assays, such as the dopaminergic cell lines (i.e., SH-SY5Y cell line or Lund human mesencephalic (*LUHMES*) neuronal cells), human stem cell lines derived from different cell types, and human-induced pluripotent stem cell (hiPSC)-derived neuronal cultures and brain organoid^152–154^ carrying known genetic risk factors, to study acute and developmental toxin-induced neurotoxicity. Quantitative high-throughput screening systems, for instance the Tox21 robotic qHTS system, can test up to one million samples per week^155^.

Testing toxins (in isolation or as mixtures) in concentrations that fall into the range of levels that are found in the human brain. The readouts in the neuronal systems involve oxidative stress, inhibition of mitochondrial activity, dysfunction of lysosome, cell viability, apoptosis and α-synuclein aggregations or other related pathways^145,146^. Furthermore, not only the neuronal system, but also the glial activation and inflammation markers should be measured in the organoids. The toxins that are found to compromise cell or organoid functions will subsequently be tested at tier 3.

### Tier 3: in vivo research

Move to *C. elegans* and *D. melanogaster* as models, which are invertebrate model systems that have been demonstrated to be able to express key aspects of PD. In addition, the *Danio rerio* can be used, a model with invertebrate-like advantages coupled with its vertebrate nature. Importantly, the use of these 3 R systems is proposed as a simple screening tool to bin chemicals that are human toxicants^156^ and previous work has proven their value in the assessment of pesticide-induced (developmental) neurotoxicity^108,109,157,158^. Using *C. elegans* in toxicity screening has demonstrated similar predictions of LD50s as rat and mouse^159,160^. Toxins can be screened for adverse effects in nematodes and fish including reproduction, growth, development, mobility and survival, thus highlighting the fact that these 3 R systems are suitable bridges between in vitro assays and mammalian toxicity screening. Additionally, neurodevelopment key events and adverse outcomes can be explored, including those that model dopamine-dependent behaviours (i.e. uncoordinated movement and growth, impaired touch response and lower basal slowing), a shorter lifespan, degeneration of dopamine cells and the formation of Lewy bodies like-structures in the brains of these animal models^161,162^. Exposures can vary in duration and time of onset (i.e., different life stages), whilst being undertaken in liquid media to aid high throughput, with the use of solid media allowing detailed descriptions of behaviour to take place^109,163^.

Toxins can best be tested using *D. melanogaster* at TD50 in feeding medium for 7 to 14 days^124^. The readouts in these models involve a major locomotor defect (i.e. staying at the bottom of vials and not appearing to coordinate the legs normally), a shorter lifespan, degeneration of dopamine cells and the formation of Lewy bodies like-structures in the brains of these animal models^124,164^. Zebrafish larvae are well suited to high throughput screening of toxins, with the response to light, sound and touch possible^165^. For juvenile and adult zebrafish, additional readouts include behavioural deficits (i.e., reduced spontaneous swimming and velocity), a shorter lifespan, degeneration of dopamine cells and the formation of alpha-synuclein accumulations like-structures in the brains of these animal models^137,138^.

In general, nematodes, fish and fruit fly models are amenable for large-scale chemical screening, thus providing a fast and relatively cheaper ways for a broad-spectrum insecticide, piscicide and pesticide screening. One significant advantage to these model systems, is using them to test mixtures of toxins, which are more representative of the environment. Is not feasible to test the effects of toxic mixtures in mammals as the large number of toxic chemical combinations require a large number of animals for testing. To this end, *C. elegans* is a valuable alternative that can be used to assess mixture toxicity and prioritise those of most concern^166–168^ which enables more directed studies to be undertaken in higher models. On the other hand, an inevitable drawback is lethality to those models, and the fact that some organ systems are not present (i.e., no circulatory system in *C. elegans*). However, this does not detract from the fact that these models can make a significant contribution to the evaluation of toxins on neurodegenerative disease onset and progression. The toxins that are found to show PD-related readouts in these models will subsequently be tested at tier 4 in concentrations that fall into the range of exposure in humans.

#### Tier 4

Finally, use experimental animals including mice and rats to study the effects of environmental factors-induced toxicity. The advantage of mouse models is that a large array of transgenics is available to study the contribution of genetic factors to PD risk. Rats, on the other hand, are more suited for elaborative behavioural and particularly cognitive assessments, as they show a broader range of behaviours and have cognitive capacities that are more advanced compared to those of mice^169^. Making use of the advantages of mice and rats we can generate high quality, human relevant, animal data for new chemicals. The approach can be projected to substances that are already on the market but get ‘flagged’ in lower tier testing strategies or new chemicals.

We suggest to start screening at tier 4 with the use of mice and rats. Expose the animals at a young adult age and old age orally to relevant concentrations of the toxins (single or multiple toxins) through oral gavage or by adding the toxin to the drinking water or by inhalation for minimal stress effects. The aging and toxin accumulation into neuromelanin cells over years are well studied^170,171^. We therefore also propose to include aging rodents (greater than 1 year old). The dosage and the minimum numbers of each sex animal needed per group can reference the Organisation for Economic Co-operation and Development Guidelines for the testing of Chemicals^172^. Subject the two species acutely, 28 days, 90 days and two year after toxin exposure to a behavioural test battery assessing motor activity (open field test for exploratory behaviour, rotarod test for motor coordination, the Catwalk test to evaluate gross motor function, the pellet grasping test to measure fine motor function, the grip test to measure muscle strength and finally the apomorphine-induced rotation test to specifically detect dopaminergic circling deficits). Additionally, use an automated home cage (e.g. Phenotyper, Noldus Information Technology) to assess day/night activity patterns and anxiety (e.g. light spot test^173,174^), as well as cognition (e.g. cognition Wall to measure reversal learning^174^). Finally, sacrifice part of the animals at each time point to assess dopamine neuron degradation in the substantia nigra through tyrosine hydroxylase (rate-limiting enzyme in dopamine synthesis), and norepinephrine neurons in the locus coeruleus through tyrosine hydroxylase, dopamine β-hydroxylase (rate-limited enzyme in norepinephrine neurons) immunostainings, and Lewy body-like formation through α-synuclein immunostainings.

We suggest to start screening at tier 1 with the use of high-throughput cell-based techniques all the way to animal models such as rodents at tier 4. The idea of the test strategy, and thus of the Fig. 1, is to provide a series of ‘filtering’ steps to select substances with a potential to induce PD. Through the sequential steps the testing becomes more specific and detailed. Starting with a large number of substances, in line with the idea to make animal experiments under 3 R principle (Replacement, Reduction and Refinement).Non-human primates are the animal species most close to humans and their brain structure is more alike to that of humans compared to mouse and rat brains. However, their use is ethically constrained. Therefore, non-human primates are not included in the test strategy.

## Conclusion

The number of people with PD has doubled to over 6 million between 1990 to 2015, and this number is projected to double again to over 12 million by 2040. Epidemiological, animal, neuropathological data strongly suggest that there are associations between PD risk and exposure to toxins, specifically rotenone, paraquat, organochlorine compounds,and metals. However, over the past half century, tens of thousands of chemicals have become commercially available and are now widely being used worldwide. A particular concern is that current internationally applied neurotoxins screening procedures inadequately identify the PD risk of any given toxin, and do not assess the risk following exposure to commonly occurring mixtures of different toxins. Because toxins in humans display their PD risk after a relatively long period of exposure, it will take years for us to identify the risk and it will take even longer for legislative actions banning or regulating these highly pervasive neurotoxic substances. While model organisms, such as worms, fish and flies are used to understand the basis of toxicity, these established methods can be easily leveraged into PD research. The advantage of utilising such (in)vertebrate animal models is that they are well suited for high-throughput testing. To better understand the interactions between environmental, genetic and neurobiological effects of the disease and to follow up on eliminating the use of chemicals known to increase the risk of PD, we argue that a well-controlled in silico, in vitro and in vivo screening platform is essential to recognize PD-related environmental risk factors.
